# Pathological alterations and COHb evaluations as tools for investigating fire-related deaths in veterinary forensic pathology

**DOI:** 10.3389/fvets.2024.1396540

**Published:** 2024-05-21

**Authors:** Giuseppe Piegari, Ilaria d’Aquino, Giovanni Valerio Salanti, Valeria Russo, Davide De Biase, Giulia Caccia, Anna Carfora, Carlo Pietro Campobasso, Orlando Paciello

**Affiliations:** ^1^Department of Veterinary Medicine and Animal Production, University of Naples Federico II, Naples, Italy; ^2^Department of Pharmacy, University of Salerno, Fisciano, Salerno, Italy; ^3^Department of Experimental Medicine, University of Campania "Luigi Vanvitelli", Naples, Italy

**Keywords:** veterinary forensic pathology, fire-related death, carboxyhemoglobin, forensic sciences, forensic pathology

## Abstract

Fire-related deaths are usually a consequence of carbon monoxide (CO) poisoning or shock from thermal injuries. In humans, high levels of carboxyhemoglobin (COHb) concentrations in the blood can support a diagnosis of CO poisoning. In veterinary medicine, few studies investigated the pathological changes and blood COHb% in fire victims, and no data are available on post-mortem changes in blood gas composition due to fire. This study aims to investigate the pathological changes and COHb levels in both animal victims of fire and cadavers experimentally exposed to fire. For this purpose, dogs were selected and subdivided into three groups. Group A comprised 9 adult dogs, and Group B comprised 7 puppies that died under fire-related conditions. Group C was represented by 4 dog cadavers experimentally exposed to heat and smoke. A complete macroscopic, histological, and COHb evaluation were performed on each animal. Animals in Groups A and B showed cherry-red discoloration, thermal-injuries and soot deposits along the respiratory tract. Animals in Group C showed thermal injuries and soot deposits limited to the upper respiratory tract. The mean COHb% values in cadavers in Group C were lower than those observed in the other groups but higher compared to the values detected before the heat and smoke treatment. These findings suggest that both pathological changes and COHb analysis are valid tools for investigating fire-related deaths in dogs. However, the increase of COHb levels in cadavers exposed post-mortem to heat and smoke highlights how the COHb analysis should always be evaluated together with macroscopical and microscopical findings to avoid significant misjudgments in investigating fire-related fatalities in veterinary forensic practice.

## 1 Introduction

Fire-related fatalities (FRFs) are defined by the UK Fire Statistics guidelines as any death “*that would not have otherwise occurred had there not been a fire*” and include “*any fatal casualty which is the direct or indirect result of injuries caused by a fire incident*” (1). Fire-related fatalities can be a consequence of a broad range of insults that can concomitantly act on the victim’s body. Indeed, ante- and post-mortem thermal injuries, traumatic lesions and carbon monoxide (CO) poisoning can all be observed in FRFs (2–8). Thermal injuries are often observed in fire-related fatalities, individually or in association with other types of injuries like ballistic, sharp force injuries, or injuries due to a blunt trauma. Thermal injuries are a consequence of exposure to heat, and their evaluation and interpretation are difficult in both human and veterinary forensic pathology (6, 7). Indeed, ante-mortem thermal wounds can be mistaken by post-mortem thermal changes because of direct and indirect fire action on animal cadavers (7, 8). Post-mortem thermal injuries can also mimic a trauma or destroy the physical evidence of a crime, making it difficult to correctly identify homicide-related injuries (7–10). The most common fire-related signs reported in the human medical literature are as follows: *soot in deep airways and the stomach, cherry-red discoloration of the body, and thermal wound* (11). These injuries can be associated with or covered by post-mortem fire-related injuries, such as *bone fractures, skin splitting and charring, muscle contraction, and retraction of the lips and tongue* (12, 13). Overall, one of the major challenges for a forensic pathologist is determining whether the victim died before or during a fire. Usually, a complete evaluation of the death scene, post-mortem macroscopic and histological findings, and toxicological analysis are necessary before making a final diagnosis (14, 15). Indeed, CO poisoning can sometimes be the main cause of death in FRFs (2). CO is a toxic, colorless, odorless, and tasteless gas that is produced whenever a substance containing carbon burns incompletely (3). CO derives from the oxidation of carbon in the presence of oxygen (3, 4). CO has a higher affinity for hemoglobin and myoglobin than oxygen, and its inhalation leads to rapid carboxyhemoglobin (COHb) formation (4, 5). COHb lowers the red blood cells’ oxygen-carrying capacity, resulting in systemic hypoxemia and hypoxia (4). The CO that binds to hemoglobin also stabilizes hemoglobin in the R-form, increases oxygen affinity at other binding spots, and leads to a concomitantly lower release in peripheral tissues (4). Consequently, COHb% in the blood is currently considered an important “*biological marker*” for the diagnosis of ante-mortem smoke exposure. The normal blood concentration of COHb is usually lower than 3%, and its increase suggests ante-mortem CO inhalation during fires (16, 17). Despite this, *in vitro* studies have demonstrated an increase of COHb% in experimentally heated blood samples in humans (18). Therefore, the possibility of post-mortem artificial changes in COHb% should be considered to avoid significant misjudgments regarding CO poisoning in human forensic pathology. In veterinary forensic pathology, a lack of medical literature, combined with a wide range of animals of interest, makes investigating fire-related deaths in animals even more difficult than in humans. Indeed, although the toxic effects of COHb have been reported in adult dogs experimentally exposed to CO (19, 20), studies on COHb evaluation in adult dogs in FRFs are limited to a few single cases and there is no reported data for puppies (21). In addition, to the best of the author’s knowledge, no studies have investigated the post-mortem effect of heat on cadaver blood gas composition in dogs. Finally, studies on ante- and post-mortem pathological changes in fire-related death in dogs are rare (21). In light of these observations, the aims of this study are as follows: (1) to assess the pathological changes in both adult dog and puppy victims of fire and cadavers experimentally exposed to heat and smoke; (2) to evaluate COHb% in the intracardiac blood and lung of adult dogs and puppies that died in fire-related conditions; and (3) to investigate COHb% in the lungs and intracardiac blood of cadavers experimentally exposed to heat and smoke.

## 2 Materials and methods

### 2.1 Study design

Twenty dead dogs were enrolled in the study and divided into three groups. Group A included 9 adult dogs (medium-sized dogs; age range, 2–6 years) that died in fire-related conditions. Group B comprised 7 puppies (age lower than 15 days) that died in fire-related conditions; Group C included 4 cadavers (medium-sized dogs; age range, 4–8 years; time since death less than 1 day) that died for causes other than fire and that were subsequently experimentally exposed to heat and smoke for 30 min. Heat and smoke were obtained via the combustion of coal and plant material placed inside a steel container. The cadavers were located inside the container at a distance of 30 cm from the heat and smoke source. Environmental temperature was evaluated using a thermometer and varied between 200 and 500°C.

The exclusion criteria for the animals enrolled in groups A and B comprised the evidence of severe pulmonary disease or systemic disorders including hyperadrenocorticism, renal disease, diabetes mellitus, neoplasia or sepsis. Table 1 summarizes the circumstance of death of the animals in Groups A and B.

**Table 1:** Circumstances of death in Group A and B animals.

Animals	Manner of death
n. 4 adult dogs (Group A) n. 6 dog puppies (Group B)	Animals died during a fire in a warehouse. The animals were trapped in the warehouse during the fire and died before the fire was put out.
n. 2 adult dogs (Group A)	Animals died during a fire in an apartment because of a gas cylinder explosion. The animals were trapped in the apartment during the fire and died before the fire was put out.
n. 2 adult dogs (Group A) n. 1 dog puppies (Group B)	Animals died during a fire in an apartment—no additional information was reported.
n. 1 adult dogs (Group A)	Animal died during a fire in an apartment because of an electrical short circuit. It was tied with a leash to a radiator and died before the fire was put out.

### 2.2 Macroscopic and histological examination

Post-mortem examination was performed on all cadavers. The forensic necropsies were performed in the necropsy room of the Department of Veterinary Medicine of the University of Naples, “Federico II,” with a standard forensic necropsy protocol previously described by Piegari et al. (22). The extent of the thermal skin injuries was evaluated according to the protocol previously described by Wohlsein et al. (7). Lung, trachea, and skin samples were collected for histopathologic examination. The samples were fixed in 10% neutral buffered formalin, embedded in paraffin, sectioned at 4 micrometers, and stained with hematoxylin and eosin (H&E) for morphological evaluation of the lesions (7, 23, 24).

### 2.3 Carboxyhemoglobin evaluation and statistical analysis

At the end of the necropsies of the Group A and B animals, intracardiac blood and lung tissues were collected for CO analysis. In Group C cadavers, intracardiac blood samples were collected both before and after the heat treatment; the ratio of COHb% before and after heat treatment was used to investigate the changes in COHb%. Lung tissue samples in Group C were collected at the end of gross examination in all assessed cases. All samples were taken using sterile instruments and frozen in freezer within 20 min. Samples were transported to the forensic toxicology laboratory of the University of Campania, “Luigi Vanvitelli,” within a week’s time. Blood and lung tissue samples were evaluated using a protocol previously described by Hartridge (25). The SPSS 20.0 package (SPSS Inc., Chicago, IL, USA) was used for statistical analysis of the data. Mann–Whitney U Test was used to assess the differences in COHb% between groups. *p*-values <0.05 were considered statistically significant.

## 3 Results

### 3.1 Macroscopic examination

GROUP A–Group A showed skin charring ranging between 20 and 90% of the total body surface and front limbs partially flexed in seven out of nine cases. Skin thermal injuries were not observed in two out of nine cases; bone fractures with severe charring and splitting of the skin were observed in one out of nine cases; and focal or multifocal skin burns were observed in two cases. Retraction of the lips and tongue was also noted in three out of nine cases. A mild to moderate cherry-red discoloration of the mucus membranes and mild to severe soot deposition in the upper and lower respiratory tract were detected in seven out of nine cases. Lungs were hyperemic and moderate to severe pulmonary edema was observed in all assessed cases. In one out of nine cases, soot deposition in the trachea lumen appeared to be mixed with a significant amount of partially undigested food material.

GROUP B–Group B showed charring of the skin ranging between 9 and 36% of the total body surface; the subcutis was hyperemic with absent to moderate cherry-red discoloration Lungs were congested with mild to moderate pulmonary edema; a mild soot deposition was observed in only three out of seven cases.

**Figure 1:**
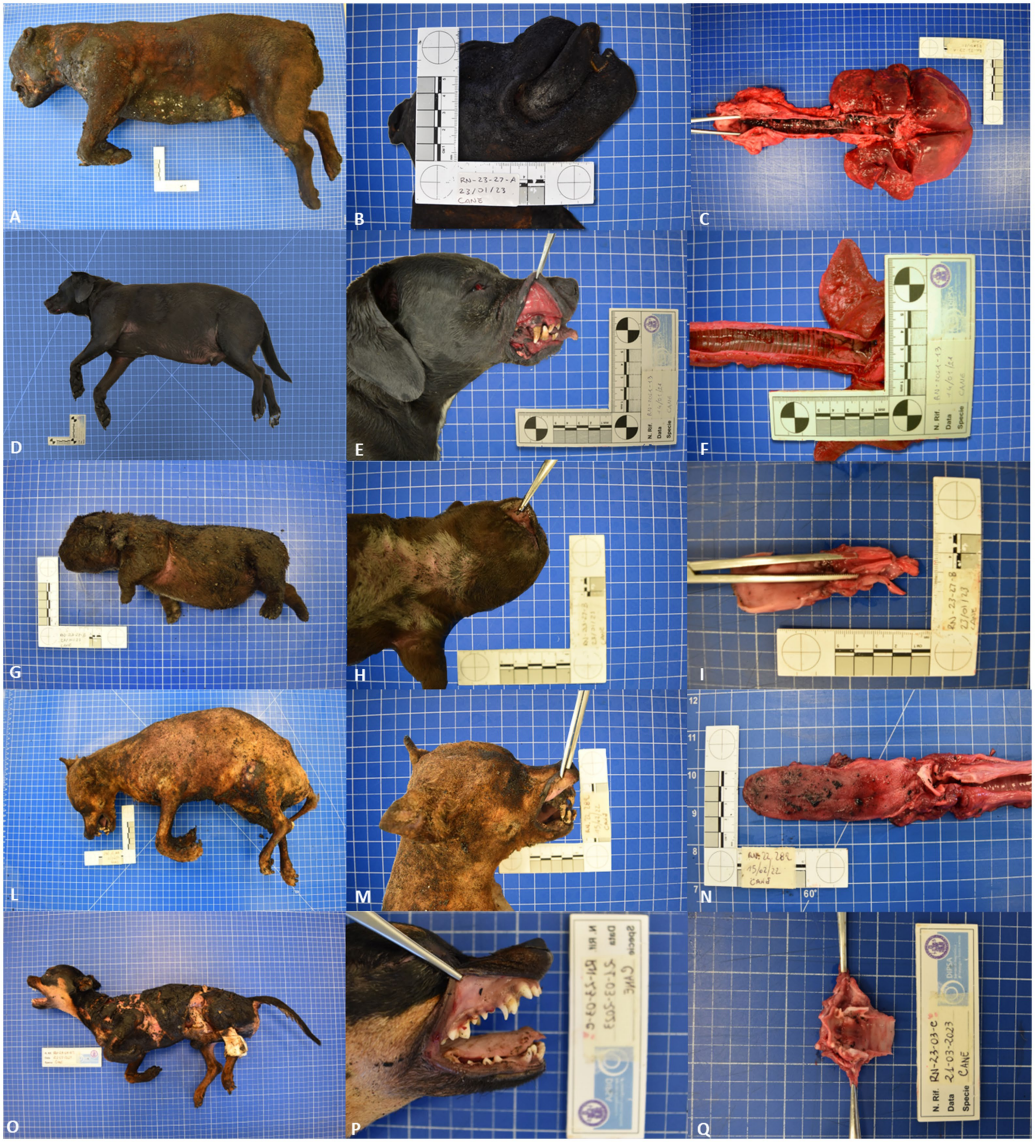
Representative pathological alterations in fire-related fatalities death cases and experimentally exposed cadavers to heat; Group A **(A)** charring of the skin and soot deposits on the surface of the skin **(B)** retraction of the lips and tongue **(C)** soot deposits on the trachea lumen; **(D)** no thermal injuries at external examination **(E)** cherry-red discoloration of the oral mucosa **(F)** soot deposits on the trachea lumen. Group B: **(G)** mild cherry-red discoloration of the postmortem lividity **(H)** reddish color of the oral mucosa **(I)** mild soot deposits on the trachea lumen. Group C: **(L)** charring and splitting of the skin with limbs partially flexed **(M)** oral mucosa showing a pale pink to white color **(N)** moderate soot deposits on the surface of the tongue **(O)** charring and splitting of the skin with limbs partially flexes **(P)** pale pink color of the oral mucosa **(Q)** moderate amount of soot deposits on the surface of the larynx.

GROUP C–Group C showed charring of the skin that appeared to be black and rock-hard. The limbs were partially flexed. Focal or multifocal skin splitting were observed in all cases. The color of the mucous membranes ranged from pale white to red; a mild soot deposition was only observed in the upper respiratory tract (the larynx, mouth, and nasal cavity) in three out of four cadavers (Figure 1; Table 2).

**Table 2:** The most common macroscopic lesions observed in the three study samples stratified by group.

Groups	Skin burns	Skin lacerations	Skin dehydration	Skin charring	Soot deposits	Cherry red discoloration	Pulmonary injuries	Bones fractures	Muscles contraction
Group A #1	–	–	–	–	X	X	X	–	–
Group A #2	–	–	–	–	X	X	X	–	–
Group A #3	–	–	X	X	X	X	X	–	X
Group A #4	X	X	X	X	X	X	X	–	X
Group A #5	X	X	X	X	X	X	X	–	X
Group A #6	–	X	X	X	X	X	X	X	X
Group A #7	–	–	X	X	X	X	X	-	X
Group A #8	–	–	X	X	–	–	X	–	X
Group A #9	–	–	–	–	–	–	X	–	–
Group B #1	–	X	–	X	X	–	X	–	X
Group B #2	–	–	X	X	X	X	X	–	–
Group B #3	–	–	–	X	–	–	X	–	–
Group B #4	-	–	X	–	X	X	X	–	–
Group B #5	-	-	X	X	-	X	X	–	–
Group B #6	–	X	X	–	–	X	X	–	–
Group B #7	–	–	–	–	–	–	X	–	–
Group C #1	–	X	X	X	X	–	X	–	X
Group C #2	–	X	X	X	X	–	X	–	X
Group C #3	–	X	X	X	X	–	X	–	X
Group C #4	–	X	X	X	–	–	X	–	X

### 3.2 Histological analysis of skin tissue samples

GROUP A–Group A showed inclination and elongation of epithelial cells in three out of nine cases; protein-rich subepidermal or intraepidermal blisters and soot deposits were observed in four cases (superficial second-degree burns). Dermal hemorrhages and necrosis were detected in two out of nine cases and a moderate to severe dermal and sub-epiderma infiltration of neutrophils were observed in two out of nine cases (second- and third-degree burns). The histopathological examination also revealed homogenization of the connective tissue with increased basophilia, dermal vacuolization, and intraepidermal or subepidermal (dermo-epidermal) detachment in five out of nine cases; a mild to moderate loss of cellular details was observed in five out of nine cases and a moderate to severe loss in four out of nine cases. Loss of cellular details were characterized by decreased stain uptake, loss of the cell borders and obscuration or disorganization of cellular structures. No histopathological alterations were observed in two out of nine cases.

GROUP B–Group B showed injuries mainly characterized by extensive homogenization of the connective tissue, increased connective tissue basophilia, and loss of cellular details.

GROUP C–Group C showed alterations mainly characterized by a mild to severe loss of cellular details, separation of the epidermis from the underlying connective tissue, vacuolization, and homogenization of the connective tissue, and increased basophilic staining. No hemorrhages or inflammatory infiltrates were observed in any of the assessed cases (Figure 1).

Table 3 summarizes the histological alterations observed in both the fire-related death cases and the experimentally exposed cadavers.

**Table 3:** Histopathological alterations observed in the study samples.

Main injuries observed in fire-related deaths (Groups A and B)	Main injuries observed in experimentally exposed cadavers (Group C)
Loss of cellular details	Loss of cellular details
Dermal vacuolization and homogenization	Dermal vacuolization and homogenization
increased basophilic staining	Increased basophilic staining
Inflammatory infiltrate, hemorrhage, and necrosis	–
inclination and elongation of epithelial cells	–
subepidermal blisters with accumulation of serum	–

### 3.3 Histological analysis of lung tissue samples

**Figure 2:**
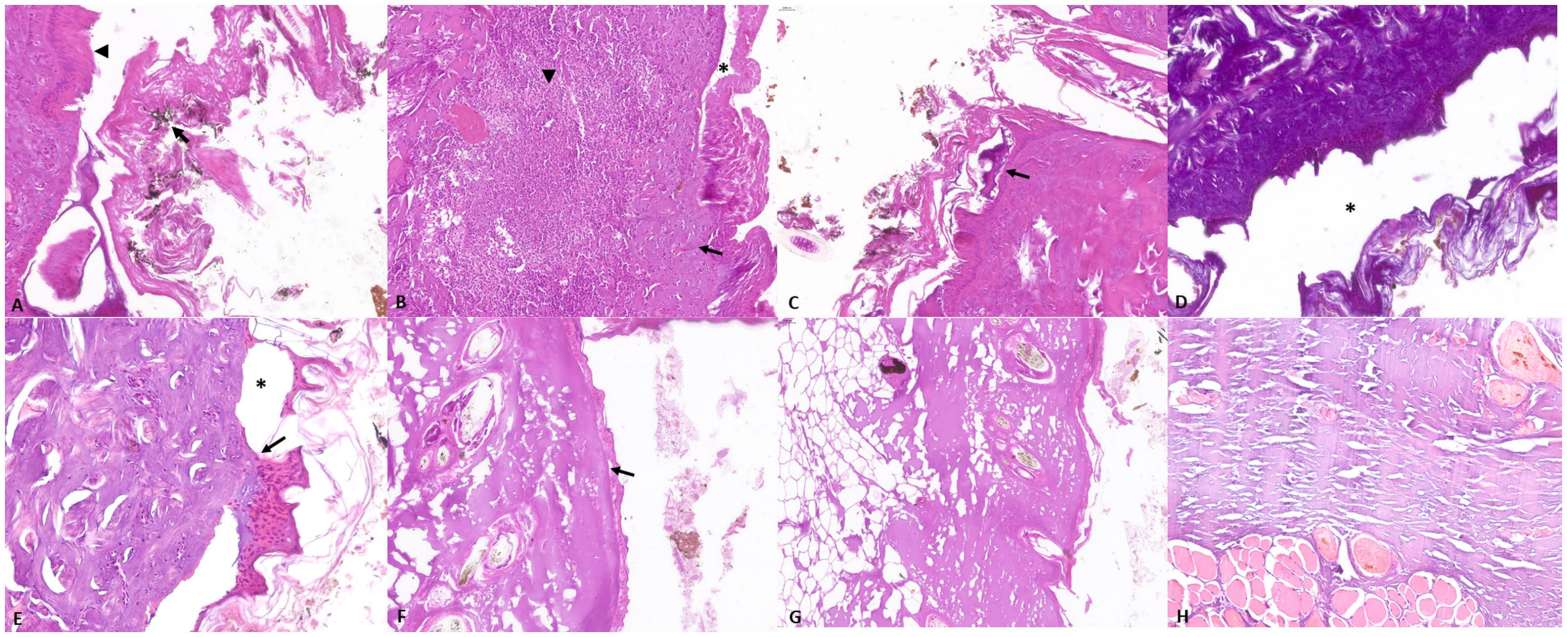
Representative skin pathological alterations in fire-related death cases (Group A and B): **(A)** soot on the surface of the skin (arrow) and elongation of epithelial cells (arrowhead) **(B)** inflammatory infiltrate characterized by neutrophilic granulocytes (arrowhead), associated with necrosis (arrow) and epithelial detachment (asterisk) **(C)** intraepidermal blister (arrow), **(D)** increase in connective tissue basophilia and dermo-epidermal and intraepidermal separation (asterisk) **(E)** dermo-epidermal separation (asterisk) and sub-epidermal blister (arrow); Representative skin pathological alterations in experimentally exposed cadavers (Group C): **(F)** loss of cellular details and dermo-epidermal separation (arrow) **(G)** dermal vacuolization **(H)** increased connective tissue basophilia.

GROUP A–Group A showed moderate to severe accumulation of pale or proteinaceous fluid within alveoli (edema) and vascular ectasia. Different amounts of amorphous black granular material were detected on the trachea, the surface of the bronchial epithelial lining, or the alveolar wall. In four out of nine cases, multifocal intra-alveolar hemorrhages and elongation of bronchial epithelial cells were also observed. In four out of nine cases, there was an extensive loss of cellular details with increased basophilic staining and in two out of nine cases, a high number of macrophages with cytoplasm rich in black particles in both the lungs and pulmonary lymph nodes (Figure 2).

GROUP B–Group B showed mild to moderate accumulation of pale or proteinaceous fluid within alveoli (edema) along with mild signs of autolysis characterized by epithelial detachment and mild loss of cellular morphology. A variable amount of black material was also detected in all cases. However, epithelial elongation was mildly evident in only one out of seven cases.

**Figure 3:**
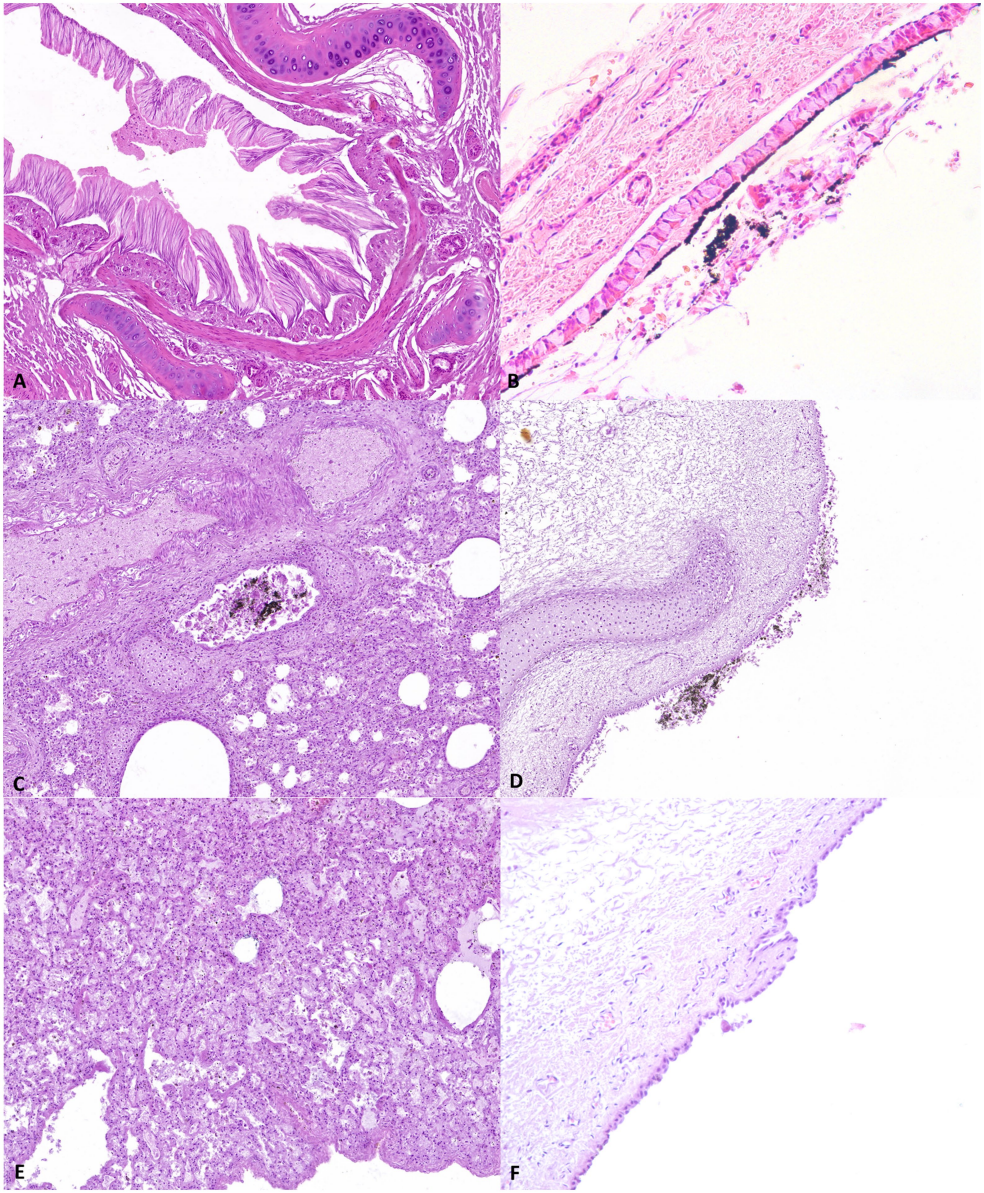
Representative pathological alterations in the study samples; Group A: **(A)** epithelial elongation **(B)** soot on the surface of the tracheal mucosa; Group B **(C)** soot in the bronchial lumen **(D)** soot on the surface of the tracheal mucosa. Group C: **(E)** pulmonary oedema **(F)** absence of soot deposits on the tracheal mucosa surface.

GROUP C–Group C showed vascular ectasia associated with a mild to severe pale or proteinaceous fluid amount within the alveoli. No amorphous black granular material or elongation of bronchial epithelial cells was observed in any of the assessed cadavers (Figure 3).

### 3.4 Carboxyhemoglobin levels in intracardiac blood and lungs

Blood COHb% in cadavers of Groups A and B ranged between 24 and 76% (mean value, 52.53 ± 20.78 SD) and 23.9 and 62.5 (mean value, 38.57 ± 12.94 SD), respectively (Table 4). An increase in COHb levels in Group C after heat treatment was observed in all the four cases in a range between 3.1 and 18.7% (mean value 12.57 ± 6.65 SD) (Table 5). Lung COHb% ranged between 31.8 and 75% in Group A (mean value, 46.51 ± 18.21 SD), 47.0 and 64.1% in Group B (mean value 51.26 ± 7.27 SD), and 4 and 13% (mean value 9.75 ± 4.03 SD) in Group C (Tables 4, 5). Statistical analysis showed a higher percentage of COHb in Group A and B blood compared with Group C. The COHb% in the lungs observed in Groups A and B was higher than the levels recorded in Group C. Finally, a comparison of the COHb% between the lungs and intracardiac blood in the same group showed no statistical differences in Groups A. However, the lung COHb% in Group B appeared to be higher than what was observed in intracardiac blood (*p* < 0.05). Tables 4, 5 summarize the percentage and the mean values of COHb for each case stratified by group.

**Table 4:** COHb% in intracardiac blood and lung samples and mean values in groups A and B.

Group	HCOb in blood	HCOb in lung	Mean in blood	Mean in lung
Group A #1	65.2%	–		
Group A #2	75.9%	69.9%		
Group A #3	76.0%	–		
Group A #4	41.4%	31.8%		
Group A #5	26.2%	33.2%	**52.53 ± 20.78 SD**	**46.51 ± 18.21 SD**
Group A #6	50.0%	75%		
Group A #7	40.9%	42.7%		
Group A #8	73.2%	40.0%		
Group A #9	24.0%	33.0%		
Group B #1	31.6%	–		
Group B #2	35.0%	48.2%		
Group B #3	28.8%	49.9%		
Group B #4	44.1%	64.1%	**38.57 ± 12.94 SD**	**51.26 ± 7.27 SD**
Group B #5	23.9%	47.0%		
Group B #6	44.1%	47.1%		
Group B #7	62.5%	–		

**Table 5:** COHb% in intracardiac blood and lung samples and mean values in group C.

Group	Increase in COHb%	HCOb% in lung	Mean in blood	Mean in lung
Group C #1	14%	10%		
Group C #2	14.5%	13%		
Group C #3	18.7%	12%	**12.57 ± 6.65 SD**	**9.75 ± 4.03 SD**
Group C #4	3.1%	4%		

## 4 Discussion

The main causes of death in FRFs are thermal injuries and CO poisoning (2). Although fire injuries have been extensively investigated in humans, few studies have characterized gross and histological lesions in animals of veterinary forensic interest (20, 21). Furthermore, no studies have investigated the differences among adult dogs and puppies who died in fire-related conditions and cadavers experimentally exposed to fire. Indeed, one of the main challenges in the investigation of cadavers recovered from a fire scene is determining whether the victim died before or during the fire. Therefore, validating a diagnostic protocol appears to be essential (1) to correctly identify the causes of death and (2) to discriminate between ante- or post-mortem exposure to fire.

### 4.1 Macroscopic and histological findings

In this study, the most common macroscopic and histological alterations observed in FRFs in adult dog cadavers (Group A) were localized to the respiratory tract, skin, and mucous membrane levels, such as *pulmonary congestion and edema; soot in the trachea, mouth, and nasal cavity; elongation of epithelial cells; cherry-red discoloration of the mucous membranes; inflammatory infiltrate; charring of the skin; and collagen homogenization*. Although some of these lesions were also found in the cadavers exposed to heat and smoke, *cherry-red discoloration* of the mucous membranes, *elongation of epithelial cells*, *dermal inflammatory infiltrate, and epidermal blisters* were alterations exclusive to animals that died in fire-related conditions. Therefore, these lesions can be considered characteristic of FRFs in adult dogs. These results agree with those reported in human forensic medicine (13). *Cherry-red discoloration* of the mucous membranes and *histopathological elongation of bronchial epithelial cells* are considered proof of respiratory activity during fires in humans (13). Similarly, *elongation of skin epithelial cells*, although described in electrical injuries, is frequently reported in fire burns (26–28). In FRFs the inhalation of the hot gas leads to “thermal damage” at the bronchiolar epithelium level, characterized by the elongation of epithelial cells (29). In addition, inhaling smoke increases COHb levels, with consequent discoloration of the mucous membranes (11, 30). Fire fumes are also responsible for the deposition of soot in the respiratory tract, which can be detected both macroscopically and microscopically (11, 29). Our results showed soot deposits in both cadavers of the Group A and those exposed post-mortem to heat and smoke of the Group C. However, relevant differences in soot localization were observed between the assessed groups; they were widespread throughout the respiratory tract in FRFs of the Group A and were limited to the upper respiratory tract in the experimentally exposed cadavers in Group C. These findings can be explained by considering the different mechanisms of smoke penetration between the two assessed groups, which are mainly due to active inhalation in live animals and post-mortem passive deposition in cadavers. Conversely, soot deposits in the lung were hardly detectable in the puppies of Group B by gross examination. However, a mild to moderate amount of soot was still observed by histopathological assessment. The puppies of Group B also showed histopathological skin injuries mostly similar to those observed in the experimentally exposed cadavers of Group C, suggesting their prevalent post-mortem nature. As frequently reported in the literature, puppies are poor regulators of their body temperature and have specific physiological characteristics that make them particularly sensitive to environmental influences (31). Furthermore, puppies respiratory rates are lower than adult dog and their response to hypoxia are minimal or absent (31); consequently, it can be hypothesized that even slight exposure to high temperatures and environmental smoke can lead to the death of the puppies. Therefore, puppies’ ante-mortem fire injuries and soot deposits may appear less evident than in adult dogs. Finally, the most common skin histopathological alterations in Group C cadavers were represented by collagen vacuolization, increased collagen basophilia, intraepidermal or subepidermal (dermo-epidermal) separation, and loss of cellular details. These changes are a consequence of rapid skin dehydration, unequal tissue shrinkage, and cellular damage due to the post-mortem action of heat on the surface of the skin (32). In addition, the lung histopathological alteration observed in group C cadavers was mainly characterized by pulmonary edema and vascular ectasia. These injuries were observed both in cadavers exposed post-mortem to fire (Group C) and in FRFs (Groups A and B); therefore, they may be considered inconclusive signs of fire-related death in dogs. Indeed, according to relevant literature (33–35), they can be also detected in a broad range of pathological conditions, such as *allergic reactions and cardiac diseases*.

### 4.2 Toxicological findings

The COHb levels detected in the intracardiac blood of animals who died due to fire ranging between 24 and 76% (mean value, 52.53 ± 20.78 SD) in Group A and 23.9 and 62.5 (mean value 38.57 ± 12.94 SD) in Group B. The high COHb percentage can be referred to the mechanism of COHb formation, which is the result of CO inhalation of the victims still alive during fires (4, 36, 37). Indeed, the physiological percentage of COHb is usually lower than 3% (16, 17), and its significant increase has been reported in animals surviving fires and in CO poisoning (19, 20, 35). Moreover, only mild increases in COHb% have been reported due to exposure to cigarettes smoke or environmental pollution and in animals with specific pathological conditions such as sepsis or respiratory diseases (38–40). Surprisingly, the comparison of the COHb levels in cadavers exposed post-mortem to fire (Group C) before and after the heat treatment showed an increase in COHb% ranging between 3.1 and 18.7% (mean value 12.57 ± 6.65 SD), suggesting a post-mortem action of the heat and smoke on animal’s blood gas composition. This result agrees with previous research studies performed under experimental conditions on humans and pigs (18, 41). These studies have shown an increase of COHb% in experimentally heated blood in humans, mainly as a consequence of a Hb-O denaturation and CO production (18). Furthermore, they reported a correlation between CO exposure and COHb% increases in stillborn piglet cadavers with multiple skin lacerations, suggesting the possibility of a post-mortem interaction between CO and Hb, resulting in COHb formation (41). In our research study, the comparison of COHb levels between intracardiac blood and the lungs also showed a different relationship between the victims died due to fire (Groups A and B). Although no statistical differences were observed in adult animals died in FRFs (Group A), higher values of pulmonary COHb were detected in puppies (Group B). In animals that die in a fire, gas exchange and COHb formation occur in the small capillaries of the lungs; these compounds only subsequently enter the bloodstream, diluting with the hemoglobin normally present in the animals’ body. Therefore, the correlation between COHb in peripheral and pulmonary blood appears to be a direct consequence of the duration of the victim’s exposure to smoke inhalation. As aforementioned, puppies have specific physiological and anatomical characteristics that make them particularly susceptible to environmental influences (31). Therefore, it is possible to hypothesize that the susceptibility of puppies to fire reduces their survival time and the consequent CO inhalation and COHb diffusion, leading to a higher COHb% in the lungs than in intracardiac blood. However, further study will be needed to confirm this hypothesis.

## 5 Conclusion

The investigation of FRFs is still a challenge in human forensic pathology as well as in veterinary forensic pathology. Our results show different pathological alterations and COHb values between animal victims of fire and cadavers exposed post-mortem to heat and smoke but died due to other causes. Our findings suggest that both histopathological alterations and COHb analysis are valid tools for investigating FRFs in dogs as well as in humans. The use of lung tissue as a complementary matrix to investigate COHb percentages in dog cadavers is also recommended. However, post-mortem increase of COHb levels in cadavers exposed to heat and smoke highlights how the COHb analysis should always be evaluated together with macroscopical and microscopical findings to avoid significant misjudgments in the investigation of FRFs in veterinary forensic pathology.

## Data availability statement

The original contributions presented in the study are included in the article/supplementary material, further inquiries can be directed to the corresponding authors.

## Ethics statement

Ethical approval was not required for the study involving animals in accordance with the local legislation and institutional requirements because Ethical review was not required because this study was conducted on animals that died spontaneously or by euthanasia due to serious health problems. None of the study animals were euthanized for research purposes.

## Author contributions

GP: Conceptualization, Data curation, Formal analysis, Investigation, Methodology, Supervision, Validation, Writing – original draft, Writing – review & editing. Id'A: Formal analysis, Investigation, Methodology, Validation, Writing – review & editing. GS: Data curation, Formal analysis, Investigation, Validation, Writing – review & editing. VR: Methodology, Validation, Writing – review & editing. DB: Methodology, Validation, Writing – review & editing. GC: Validation, Writing – review & editing. AC: Formal analysis, Investigation, Validation, Writing – review & editing. CC: Conceptualization, Supervision, Validation, Writing – review & editing. OP: Conceptualization, Funding acquisition, Investigation, Supervision, Validation, Writing – review & editing.
